# Identification of genes differentially expressed in granulosa cells from women with high vs. low ovarian responsiveness

**DOI:** 10.3389/frph.2026.1819763

**Published:** 2026-06-10

**Authors:** Alexa Medica, Matthew E. Kim, Wei-Ting Hung, Abhishek Sohni, Antoni Duleba, Kun Tan, Miles F. Wilkinson

**Affiliations:** 1 Department of Obstetrics, Gynecology, and Reproductive Sciences, University of California San Diego, La Jolla, CA, United States; 2 Institute of Genomic Medicine (IGM), University of California San Diego, La Jolla, CA, United States

**Keywords:** apoptosis, cumulus cells, differentiation, gene biomarkers, granulosa cells, *in vitro* fertilization, ovarian responsiveness, proliferation

## Abstract

**Background:**

A better understanding of the response of granulosa cells to exogenous hormone stimulation and how this impacts the complex interplay between the granulosa cells and the oocyte is crucial for the optimal management of infertile couples seeking IVF treatment.

**Methods:**

Granulosa and cumulus cells (G + CCs) from women with different degrees of ovarian responsiveness to controlled ovarian hyperstimulation (COH) were purified and used for RNA sequencing (RNA-seq) analysis and downstream bioinformatic analyses.

**Results:**

RNA-seq analysis on G + CCs purified from 16 patients undergoing hormone treatment for *in vitro* fertilization (IVF) identified 291 genes that are statistically downregulated in G + CCs from poor ovarian responders (PORs) as compared with G + CCs from normal ovarian responders (NORs). Downregulated genes are enriched for several gene ontology (GO) categories, including pro-proliferation functions (e.g., *AURKB, BUB1, CCNA2, CCNB1, CCNB2, CDK1, CDC20*, and *NEK2*) and DNA damage-response (e.g., *BCL2, BLM,* and *BRCA1*). Upregulated genes in POR G + CCs include those encoding secreted proteins with known granulosa cell roles (*ANGPTL4, IL1RL1*, and *STC1*) and genes linked to apoptosis and ovarian malignancy. Weighted gene co-expression network analysis identified 6 gene modules significantly correlated with the POR phenotype; integration with differential expression analysis revealed core genes associated with weak ovarian responsiveness, including those involved in canonical folliculogenesis (*ESR1, FSHR, HES1*, and *HEY2*), steroidogenesis (*ANGPTL4*, *GSTA1*, and *HSD17B1*), and cell-cycle regulation (*CCNA2*, *CCNB1*, *CDK1* and *FOXM1*).

**Conclusions:**

Our study (i) offers potential insight into the underlying pathophysiologic processes underlying weak ovarian responses, (ii) identifies genes that are candidates to define weak ovarian responders, and (iii) identifies signaling pathways that when modulated, could potentially be used to improve IVF outcomes.

## Introduction

*In vitro* fertilization (IVF) is commonly employed to achieve pregnancy in the context of either infertility or subfertility (1). IVF depends on controlled ovarian hyperstimulation (COH), which promotes simultaneous growth and maturation of multiple ovarian follicles. The success of COH regimens are highly variable between individuals, and thus several surrogate markers are used to estimate an individual's ovarian responsiveness to stimulation, including age, antral follicle count (AFC), and Anti-Mullerian hormone (AMH) levels (2). Despite efforts to individualize COH regimens, approximately 9%–24% of patients undergoing IVF are poor responders, with cycles resulting in retrieval of very few oocytes and low clinical pregnancy rates (2).

Granulosa and cumulus cells (G + CCs) are critical for the success of COH regimens, as they form multiple layers surrounding the oocyte, allowing them to provide both structural support and paracrine support, the latter by secreting key factors, including estradiol, inhibin, and growth factors that coordinate follicle growth and oocyte maturation (3–5). Granulosa cells are broadly classified into two functionally distinct subpopulations based on their spatial organization within the follicle: mural granulosa cells, which line the follicle wall and are primarily responsible for steroidogenesis, and cumulus cells, which directly envelop the oocyte and form the cumulus-oocyte complex. Following ovulation, mural granulosa cells, and cumulus cells, undergo luteinization, a process of terminal differentiation driven by the LH surge, whereby they cease proliferating, undergo dramatic morphological reorganization, and transition to progesterone-secreting luteal cells of the corpus luteum (6).

A better understanding of the G + CCs response to exogenous hormone stimulation and how this impacts the complex interplay between the GCs and the oocyte is crucial for the optimal management of infertile couples seeking IVF treatment. Generally, a COH regimen includes: Menopur (a combination of follicle stimulating hormone [FSH] and luteinizing hormone [LH) with Follistim (FSH), the latter of which is used to increase the FSH/LH ratio. Some patients also receive clomiphene citrate, a selective estrogen receptor modulator that increases endogenous FSH and LH production. FSH is an essential component of all COH regimens because this hormone induces the expansion of granulosa cells, which is necessary for meiotic maturation and the acquisition of developmental competence of the oocyte through the mitogen-activated protein kinase (MAPK) pathway (7). Emerging evidence suggests that granulosa cell sensitivity to FSH treatment, as evidenced by a higher percentage of antral follicles that respond to FSH, not only increases the number of oocytes retrieved, but also appears to significantly increase embryo quality as demonstrated by increased pregnancy rates in more responsive individuals (8).

The molecular mechanisms responsible for a poor ovarian response to COH are not well understood. Desai et al. showed that poor ovarian responders (PORs) have altered expression of specific mRNAs and allelic heterogeneity in their granulosa cells that may be contributing to the pathologic phenotype (9). This study identified specific genotypes that consistently predicted a poor ovarian response to COH, and showed that *FSHR* mRNA expression levels in granulosa cells from women with the G/G-Asn/Ser genotype were significantly lower than women with other genotypes (9). Another study found that both GDF9 and BMP15 were downregulated in both the follicular fluid and the granulosa cells from PORs, both of which were predictive of poor oocyte quality and IVF outcomes (10). Although these studies provide some insight into the possible mechanisms underlying a poor response to COH, they did not use whole transcriptome analysis to provide an unbiased view of the heterogenous molecular response of granulosa cells to hormone treatment.

Micro (mi) RNAs may also play a role in the pathophysiology of PORs. miRNAs are small (21–25 nucleotides) non-coding RNAs that function to suppress expression of their target mRNAs in a sequence-specific manner. Genome-wide studies have identified shifts in expression of miRNAs during various ovarian processes, and functional studies have identified miRNAs that have roles in specific ovarian processes, including granulosa cell apoptosis and proliferation, as reviewed by Zhang et al. (11). However, to our knowledge, studies have not examined whether specific miRNAs are dysregulated in POR vs. NOR G + CCs.

In the present study, we performed RNA sequencing (RNA-seq) analysis on G + CCs from normal and poor responders to obtain an unbiased view of differential expression of both miRNAs and protein-coding genes. This analysis revealed a gene signature that characterizes patients with poor ovarian responsiveness. It also offered insights into the underlying pathologic processes underlying PORs, and identified signaling pathways that, when modulated, could potentially be used to improve IVF outcomes for POR patients.

## Methods

### Human samples

The experiments with human material were approved by the UCSD Human Research Protections Program (HRPP) council. Informed consent was obtained from all the human subjects. The institutional review boards at the University of California, San Diego approved this study under IRB#151487.

### Study population

This analysis was conducted using follicular aspirate samples from 16 patients (23 to 44 years-old) undergoing IVF treatment for infertility at Reproductive Partners IVF Center/University of California, San Diego (UCSD). All specimens were obtained from patients (between 23 and 44 years old) undergoing assisted reproductive technology (ART) procedures at our center. Patients were included if they were undergoing ART and consented to research use of their surplus biological material. Exclusion criteria included lack of consent, incomplete clinical data, and/or cancelled cycles prior to oocyte retrieval. Participants were recruited between February 2016 and November 2017. Baseline laboratory values, including AMH, FSH and E2, were obtained for each patient. Because AMH is not cycle dependent (12), it was collected prior to IVF cycle initiation. FSH levels were determined between days 2 and 5 of the menstrual cycle. Estradiol levels were determined either daily or every other day during the IVF cycle. Siemens immunoassays were used for all laboratory tests. Supplementary Table S1 provides the following infertility diagnoses for the cohort: male factor infertility (*n* = 5), tubal factor (*n* = 1), unexplained infertility (*n* = 4), genetic disorders (*n* = 1), sex selection for family balancing (*n* = 1), oocyte donors (*n* = 2), and fertility preservation (*n* = 2). In most cases, embryos generated by IVF/ICSI were frozen for subsequent embryo transfer (*n* = 14); in two cases of fertility preservation, the oocytes were frozen before IVF/ICSI. No cancelled cycles were included.

### Collection of follicular fluid

Patients undergoing egg retrievals at Reproductive Partners IVF center in La Jolla, CA consented to collection and use of follicular fluid for research purposes. Briefly, patients underwent a synchronization phase using either oral estradiol or combined oral contraceptive pills, followed by initiation of ovarian stimulation with recombinant FSH and/or hMG. Gonadotropin dosing was individualized based on age, ovarian reserve markers (AMH and/or AFC), and prior cycle response, with adjustments made according to follicular growth and serum estradiol levels during monitoring visits. In selected patients with diminished ovarian reserve or prior suboptimal response, clomiphene citrate was provided to reduce gonadotropin requirements. Final oocyte maturation was triggered using hCG or a dual trigger (hCG + GnRH agonist) when clinically indicated. Oocyte retrieval was performed 35.5 h later under transvaginal ultrasound guidance. The follicular fluid was collected via transvaginal ultrasound-guided aspiration using an oocyte aspiration needle into test tubes, which were then emptied into petri dishes. The embryologist then removed the oocyte from the surrounding fluid, and the remaining fluid that would normally be discarded was pipetted into tubes that were placed on ice and transported to the Sanford Consortium Research Building in La Jolla (within 1 h) for isolation and purification of the G + CCs. Follicular fluid was pooled from follicles within a single cycle.

### Purification of G + CCs

G + CCs were isolated by Percoll and MACS sorting as previously described (13, 14). In brief, the follicular fluid aspirate (a mix of follicular fluid, G + CCs, and blood cells) was filtered for cellular debris using a 0.45 µm cell filter and centrifuged for 20 min at 400 *g* and 4°C to pellet all of the cells. The cell pellet was resuspended in phosphate buffered saline (PBS) wash and layered onto 50% Percoll solution. Cells were then spun for 40 min at 850 *g* at 4°C to pellet the red blood cells (RBCs). The cells at the interface, consisting predominantly of G + CCs and leukocytes, were then carefully removed and washed 3x with PBS (centrifuged at 400 *g* for 5 min at 4°C) to remove the Percoll. The pellet was incubated with antibodies against CD235a/Glycophorin A (RBC marker) and CD45 (a leukocyte marker) for 20 min at 4°C. After washing with PBS and centrifuging for 5 min at 300 *g* at 4°C, the samples were incubated with magnetic microbeads covalently bound to CD45 monoclonal antibody for 20 min at 4°C. After washing with PBS and resuspending in MACs buffer, samples were run through the MACS column following manufacturer's instructions, and the CD45-/CD235a- samples were collected for RNA isolation and sequencing. Cells were snap frozen in liquid nitrogen to preserve RNA integrity.

### RNA-seq, small RNA-seq, and data analysis

Total RNAs were isolated using Zymo Direct-zol, and small RNAs were isolated using the Invitrogen mirVana kit following manufacturer's recommended instructions. RNA quality was assessed using an Agilent Bioanalyzer prior to library preparation, and all samples demonstrated high integrity with RIN values >8. RNA-seq libraries were prepared using the Truseq v2 library kit and small RNA-seq libraries were prepared using the NEBNext small RNA library kit.

RNA-seq libraries were sequenced with an Illumina HiSeq 4,000 platform at the UCSD Institute for Genomic Medicine (IGM) core. RNA-seq analysis was performed as previously described (15–19). Briefly, raw sequencing data was filtered for quality and mapped to the human genome (GRCh38, version 95) using the STAR program (2.5.2b). Exon counts were aggregated for each gene to build a read count table using featureCounts. Differentially expressed genes (DEGs) were defined using DESeq2 (20) using the following threshold: |Log2FC| > 1 and *q*-value <0.05. The database for annotation, visualization and integrated discovery (DAVID) v6.8 was used for gene ontology (GO) analysis; Kyoto Encyclopedia of Genes and Genomes (KEGG) was used for signaling pathway analysis (21, 22). Weighted gene co-expression network analysis (23) was used to identify gene modules significantly correlated with POR (*p* < 0.05). Within each POR-associated module, intramodular connectivity (module membership, kME) and gene significance (GS) were quantified. Genes were ranked by their combined score (kME × GS), reflecting both centrality within the co-expression network and association with the POR trait; those in the top decile of each module were defined as “core genes”. To enhance biological interpretability, core genes were intersected with the differentially expressed genes identified in the analysis described above, and the overlapping genes were considered to be the most physiologically relevant.

For small RNA-seq, we used the Genboree exceRpt pipeline (24) for trimming, quality control, alignment, and reads counting. We then used DESeq2 for differential expression analysis, as described above.

### qRT-PCR analysis

As previously described (25, 26), quantitative reverse transcription (qRT)-PCR analysis was performed using the iScript™ cDNA synthesis kit (Bio-Rad), followed by PCR amplification using SYBR Green (Bio-Rad) and the ΔΔCt method (with ribosomal L19 for normalization). Results were from at least three independent replicates. Statistical significance was determined using the paired Student's *t*-test.

### Statistical analysis

Continuous variables were assessed for normality using the Shapiro–Wilk test. Homogeneity of variance was statistically analyzed using the Levene's test. Normally distributed variables were compared between the NOR and POR groups using an unpaired two-tailed Student's *t*-test. A *p* < 0.05 value was considered statistically significant. Statistical analyses and graph generation were performed using GraphPad Prism version 10.

## Results

### Characteristics of poor and normal ovarian responders

Our analysis was conducted on follicular aspirate samples from 16 patients, ages 23 to 44 years old, undergoing hormone treatment for IVF at Reproductive Partners IVF Center in San Diego, California. Participants were recruited between February 2016 and November 2017. The number of oocytes retrieved ranged from 4 to 29 (Supplementary Table S1). Seven patients were classified as POR (≤10 oocytes retrieved), and the remaining patients were classified as NOR. While PORs are often classified as those with ≤5 oocytes retrieved (27), we observed that patients retrieving 6–10 oocytes exhibited clinical and endocrine characteristics highly similar to those with ≤5 oocytes (Supplementary Figure S1). Based on this convergence of published evidence and cohort-specific data, we adopted a more biologically coherent cutoff (≤10 oocytes retrieved) to define POR in this study. This approach improved phenotype homogeneity and enhanced the power to detect transcriptomic differences relevant to impaired ovarian responsiveness.

**Figure 1:**
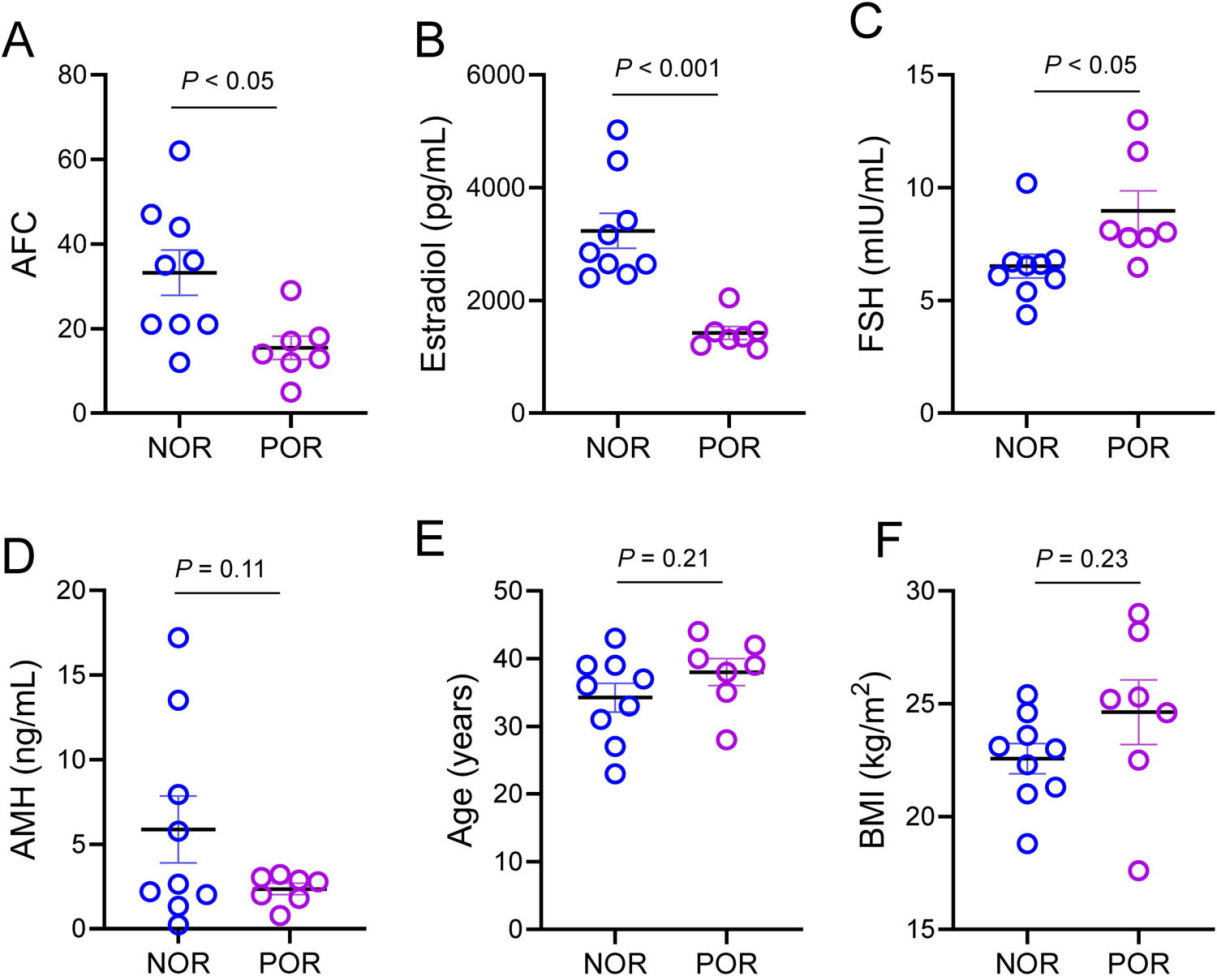
Women in our cohort with a weak ovarian responsiveness to COH have statistically reduced AFC, estradiol, and FSH levels. **(A)** Antral follicle count (AFC), **(B)** Estradiol level (pg/mL) on day of hCG trigger, **(C)** follicle-stimulating hormone (FSH) level, **(D)** Anti-Mullerian hormone (AMH) level, **(E)** Age, and **(F)** Body mass index (BMI) (kg/m^2^).

The clinical characteristics of the POR and NOR groups are presented in Supplementary Table S1. The POR group had significantly lower antral follicle count (AFC) at the start of the IVF cycle as compared to the NOR group (Figure 1A; *p* < 0.05), consistent with past reports showing weak responders tend to have lower AFC than NORs (28, 29). Likewise, the POR group had statistically lower estradiol (E2) levels on the day of hCG treatment than the NOR group (Figure 1B). This is consistent with current clinical practices that use E2 level as one of the primary predictors for both the timing of the hCG trigger and as an estimate of the number of mature oocytes (30). The rationale behind this is G + CCs convert androgens produced by thecal cells to estradiol via aromatase; thus, with increased follicular recruitment and development, estradiol levels increase (31). The third parameter that was significantly lower in the POR group was FSH level (Figure 1C), which is consistent with the critical role of FSH signaling in ovarian responsiveness, including granulosa cell proliferation, differentiation, estradiol production, and maturation of ovarian follicles (32, 33). Although AMH levels trended lower in the POR group relative to the NOR group (Figure 1D), this difference did not reach statistical significance. Age and BMI were similar between two groups (Figures 1E,F).

### Genes statistically differentially expressed in G + CCs from NOR vs. POR patients

**Figure 2:**
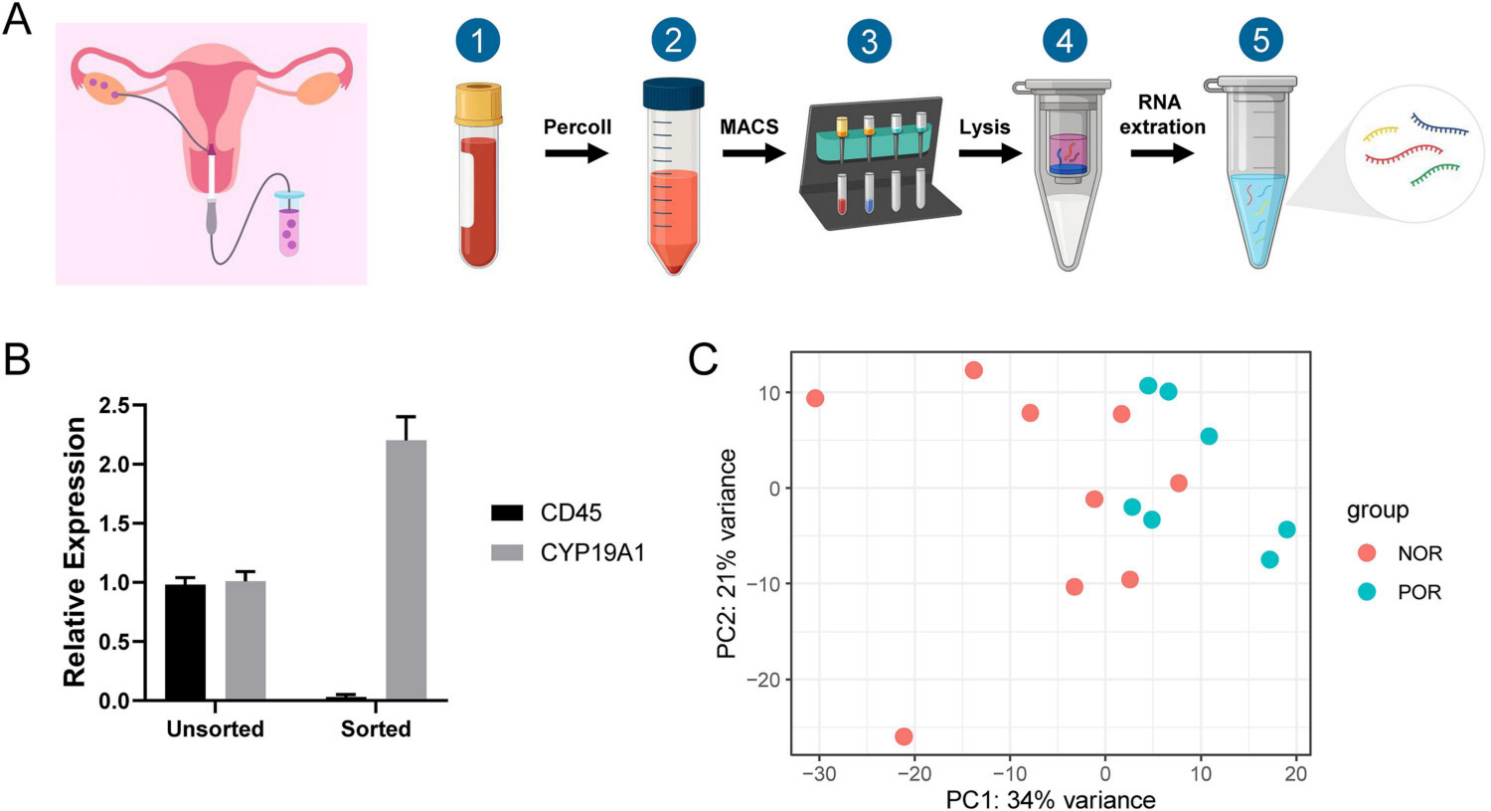
Sample preparation and RNA sequencing (RNA-seq) analysis. **(A)** Follicular aspirates were obtained using the standard egg retrieval technique. The G + CCs were purified from the aspirates (see Materials and Methods) and then incubated with antibodies against CD235a (RBC marker) and CD45 (leukocyte marker). The cell suspension was then run through magnetic-activated cell sorting (MACS) column, and the CD45-/CD235a- population was used for RNA isolation and sequencing. **(B)** qPCR analysis showing enrichment of the GC marker, CYP19A1, and reduction of CD45 expression in MACS-sorted cells compared to non-sorted cells. **(C)** PCA showing clustering of NORs (in red) and PORs (blue) by their gene expression profiles.

To identify genes differentially expressed in G + CCs from patients with different magnitudes of ovarian responsiveness, we obtained follicular aspirates (pooled from follicles within a single cycle) from the 16 patients (described above) after hormone treatment. To remove red blood cells and leukocytes, we performed magnetic-activated cell sorting and collected CD45-/CD235a- cells (Figure 2A), as reported previously (14). qPCR analysis showed that these CD45-/CD235a- cells are highly-statistically enriched for the GC marker, *CYP19A1*, and are statistically de-enriched for the leukocyte marker, *CD45* (Figure 2B).

**Figure 3:**
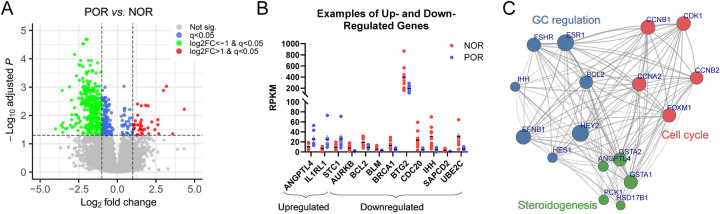
Identification of genes differentially expressed in granulosa cells from PORs vs. NORs. **(A)** Volcano plot showing significantly differentially expressed genes, as identified by RNA-seq analysis. Each point represents a single gene, plotted in terms of its magnitude of altered expression (*x*-axis, log2 fold-change) and statistical significance (*y*-axis, –log10 adjusted *p*-value [*q*-value). Genes meeting the threshold for statistically significant differential expression (*q* < 0.05) are shown in color: those upregulated by >2-fold are highlighted in red; those with smaller but significant changes (≤2-fold) are shown in blue; those downregulated by 2-fold are highlighted in green. Non-significantly regulated genes are displayed in gray. **(B)** Representative genes significantly up- or down-regulated in PORs compared with NORs. Values shown are reads per kilobase million (RPKM) of 16 analyzed samples. Error bars represent mean  ± SEM. **(C)** Co-expression network of POR-associated core genes showing functional clustering of granulosa cell regulators, steroidogenic genes, and cell-cycle drivers, with node size proportional to hub strength (kME × GS).

We performed RNA-seq analysis on these 16 purified G + CCs samples (Supplementary Figure S2). Principal component (PC) analysis showed that the POR samples clustered together, and tended to be separate from the NOR samples (Figure 2C). To define statistically differentially expressed genes between the G + CCs samples from the POR and NOR responders, we used the DESeq2 program (34), which identified 291 downregulated and 24 upregulated genes in the POR group relative to the NOR group (fold-change >2, *q* < 0.05; Figure 3A and Supplementary Table S2).

Notable statistically upregulated genes include *STC1* and *ANGPTL4* (Figure 3B), which encode secreted proteins that have been shown to promote folliculogenesis and inhibit granulosa cell proliferation, respectively (35, 36). Another upregulated gene—*IL1RL1* (Figure 3B)—encodes the receptor for the cytokine IL-33, which is dysregulated in cumulus cells from patients with diminished ovarian reserve (37).

The largest group of statistically downregulated genes are those involved in cell cycle regulation and progression. Of the 291 statistically downregulated genes, 52 are known to be involved in proliferation. Among these downregulated proliferation-promoting genes are those encoding UBE2C, an essential co-factor for the anaphase promoting complex (APC) (38), CDC20, a regulatory protein that is required for full ubiquitin ligase activity of the APC (39), and SAPCD2, which promotes cell-cycle progression and activates signaling pathways, including the MAPK and WNT pathways (40) (Figure 3B).

Another large class of statistically downregulated genes are those encoding proteins involved in DNA damage responses. In total, we identified 19 genes in this class (Table 1). Among these DNA-damage response genes, *BCL2, BLM, BRCA1,* and *BTG2* have known roles in granulosa cells (41–43, 45, 46, 48) (Figure 3B). Another downregulated gene of interest is *IHH* (Figure 3B), which encodes a secreted signaling protein that has been shown to recruit theca cells, thus its downregulation may result in impaired steroidogenesis (61).

**Table 1:** DNA-damage response genes downregulated in GCs from weak ovarian-response patients.

Gene	Gene symbol	Log2 fold change	Adjusted *p*-value	DNA damage functions	Granulosa cells and female fertility	Apoptosis	Malignancy
BCL2 apoptosis regulator	BCL2	−1.119	0.0046	Mitochondrial protein that modulates DNA repair	Expressed in GCs where it helps regulate cell death and follicle survival (41, 42).	Anti-apoptotic	Oncogene
BLM RecQ like helicase	BLM	−1.174	0.0456	DNA helicase that maintains genome stability	Promotes GC proliferation and survival (43).		Tumor suppressor
BRCA1 DNA repair associated	BRCA1	−1.318	0.0305	DNA repair protein that repairs double-stranded DNA breaks	Suppresses GC apoptosis and aromatase expression; expression in GCs suppresses E2 levels (44–46).	Apoptotic or anti-apoptotic	Tumor suppressor
BRCA1 interacting helicase 1	BRIP1	−1.458	0.0097	DNA helicase that repairs double-stranded DNA breaks		Apoptotic	Tumor suppressor. Missense mutations associated with ovarian cancer (47)
BTG anti-proliferation factor 2	BTG2	−1.030	0.0267	Mitochondrial protein that accelerates double-stranded DNA repair	Highly expressed in GCs and promotes their survival and proliferation (48).	Apoptotic	Tumor suppressor
Cellular inhibitor of PP2A	CIP2A	−1.350	0.0080	Maintains chromosomal integrity	Associated with female fertility (49)	Anti-apoptotic	Oncogene
Cyclin dependent kinase 1	CDK1	−1.966	0.0021	Serine/threonine kinase checkpoint protein that ensures DNA damage is repaired		Anti-apoptotic	Oncogene. Overexpressed in many tumors, including granulosa cells tumors (50)
Denticleless E3 ubiquitin protein ligase homolog	DTL	−1.653	0.0029	Substrate receptor for E3 ubiquitin ligase homolog that maintains DNA stability by preventing DNA re-replication		Anti-apoptotic	Oncogene
F-box protein 5	FBXO5	−1.051	0.0183	F-box checkpoint protein that promotes DNA integrity		Anti-apoptotic	Oncogene. High expression in ovarian tumors predicts poor prognosis (51)
H2A.X variant histone	H2AX	−1.144	0.0009	Histone protein that recognizes double-stranded DNA strand breaks	Expressed in ovary, particularly GCs (45)		Tumor suppressor. Often upregulated in ovarian cancer (52)
Minichromosome maintenance complex component 7	MCM7	−1.038	0.0025	DNA helicase essential for DNA replication and genome stability		Anti-apoptotic	Oncogene. Overexpressed in high-grade serous ovarian cancer (53)
Minichromosome maintenance 10 replication initiation factor	MCM10	−1.734	0.0098	DNA helicase activator essential for DNA replication and genome stability	Required for oogenesis in flies (54)		Oncogene. Prognostic biomarker correlated with immune checkpoints in ovarian cancer (55)
PCNA clamp associated factor	PCLAF	−1.885	0.0029	PCNA-associated factor involved in cell-cycle regulation and DNA damage responses			Oncogene. Overexpressed in high-grade serous ovarian cancer (56)
DNA polymerase theta	POLQ	−2.013	0.0041	DNA polymerase/DNA repair enzyme		Anti-apoptotic	Oncogene
RAD51 associated protein 1	RAD51AP1	−1.426	0.0032	DNA repair protein that maintains DNA integrity	Expressed in ovary, particularly GCs (45)	Anti-apoptotic	Oncogene. Overexpressed in ovarian cancer (57)
DNA topoisomerase II alpha	TOP2A	−2.119	0.0030	DNA topoisomerase critical for DNA repair			Oncogene. Promotes ovarian cancer cell proliferation (58)
Ubiquitin conjugating enzyme E2T	UBE2T	−1.665	0.0028	E2 ubiquitin-conjugating enzyme critical for DNA repair		Anti-apoptotic	Oncogene. Overexpression in ovarian cancer associated with poor prognosis (59)
Ubiquitin like with PHD and ring finger domains 1	UHRF1	−1.107	0.0276	DNA sensor and scaffold for DNA repair factors		Anti-apoptotic	Oncogene. Overexpressed in ovarian cells; promotes ovarian tumor proliferation and survival (60)
WD repeat domain 36	WDR76	−1.091	0.0410	E3 ubiquitin ligase that acts as a DNA damage sensor and scaffold for DNA repair factors		Apoptotic	Tumor suppressor

To identify high-confidence genes associated with POR, we performed a weighted gene co-expression network analysis. This identified 6 gene modules significantly correlated with the POR phenotype (*p* < 0.05). Within each POR-associated module, we quantified both intramodular connectivity (module membership, kME) and gene significance (GS). This allowed us to define “core genes” as those with the highest combined (kME × GS) score. Integrating these results with our differential expression analysis (POR vs. NOR) revealed that canonical folliculogenesis and granulosa-cell regulator genes (*FSHR, ESR1, HES1, HEY2, IHH, EFNB1,* and *BCL2*), steroidogenic genes (*HSD17B1, GSTA1, GSTA2, PCK1,* and *ANGPTL4*) and cell-cycle drivers involved in granulosa cell proliferation (*FOXM1, CCNA2, CCNB1, CCNB2,* and *CDK1*) as key genes and functions associated with weak ovarian responsiveness (Figure 3C and Supplementary Table S3).

### Biological functions associated with POR downregulated genes

**Figure 4:**
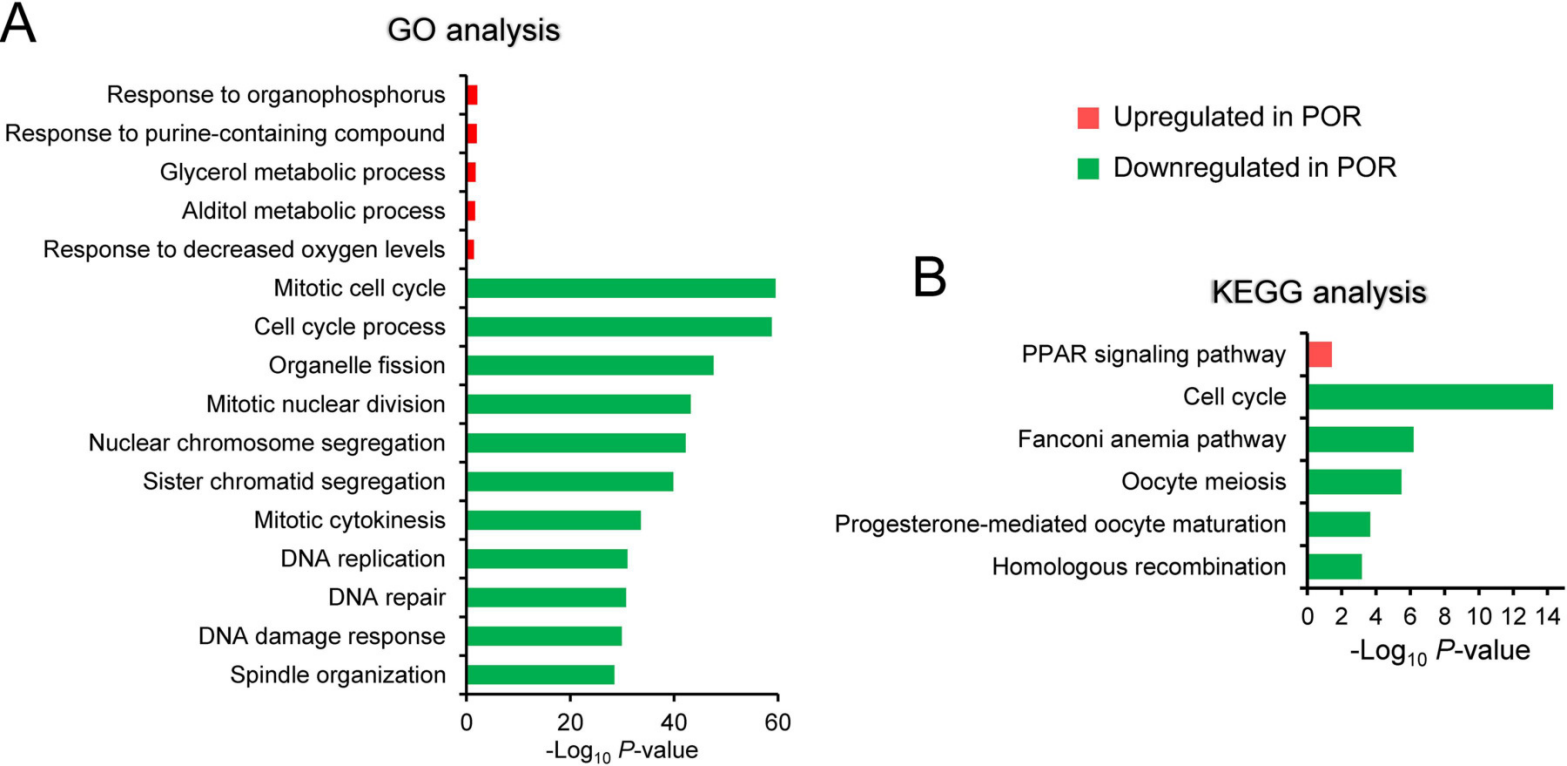
Functions statistically associated with genes statistically up- or down-regulated in GCs from women with weak ovarian responses. Gene ontology **(A)** and KEGG pathway **(B)** analyses of DEGs, as defined in Figure 3. Significantly up-regulated functional items are shown in red, and significantly down-regulated functional items are shown in green.

To identify biological functions associated with poor ovarian responsiveness, we performed GO analysis on the genes exhibiting statistically significant differences in expression between POR vs. NOR G + CCs. Consistent with our finding that many proliferation genes are downregulated in GCs from PORs (see above), GO analysis showed that the top 6 most statistically-significant functions all involve cell proliferation: “mitotic cell cycle”, “mitotic nuclear division”, “organelle fission”, “sister chromatid segregation”, “nuclear chromosome segregation”, and “cell cycle process” (Figure 4A). This was confirmed by KEGG pathway analysis, which showed that the downregulated genes are statistically enriched for proliferation-associated pathways, such as “cell cycle” and “homologous recombination” (Figure 4B). As described in the Discussion, these results raise the possibility that G + CCs from PORs tend to be less proliferative than G + CCs from NORs, and that one or more of these downregulated proliferation genes may have a causal role in this reduced proliferation.

We also found that the functional categories—“oocyte meiosis” and “progesterone-mediated oocyte maturation”—were also statistically enriched among downregulated genes in the POR group vs. the NOR group (Figure 4B). This raises the possibility that G + CCs from weak ovarian responders are less supportive of oocyte progression, a point we describe further in the Discussion.

### Biological functions associated with POR upregulated genes

The genes upregulated in G + CCs from PORs are statistically enriched for several processes, including those shown in Figure 4A. Of note, however, the statistical significance values for these GO categories are relatively low because far fewer genes were statistically upregulated (24) than downregulated (291). Interestingly, the only statistically-significant KEGG pathway category associated with the upregulated genes is the PPAR signaling pathway (Figure 4B). The 3 upregulated genes associated with PPAR signaling—*PCK1, ANGPTL4*, and *STC1*—are all known to influence cell proliferation (36, 62). SCT1 encodes a secreted glycoprotein that promotes folliculogenesis by promoting granulosa cell proliferation and steroidogenesis (36).

### miRNAs differentially expressed between POR vs. NOR G + CCs

**Figure 5:**
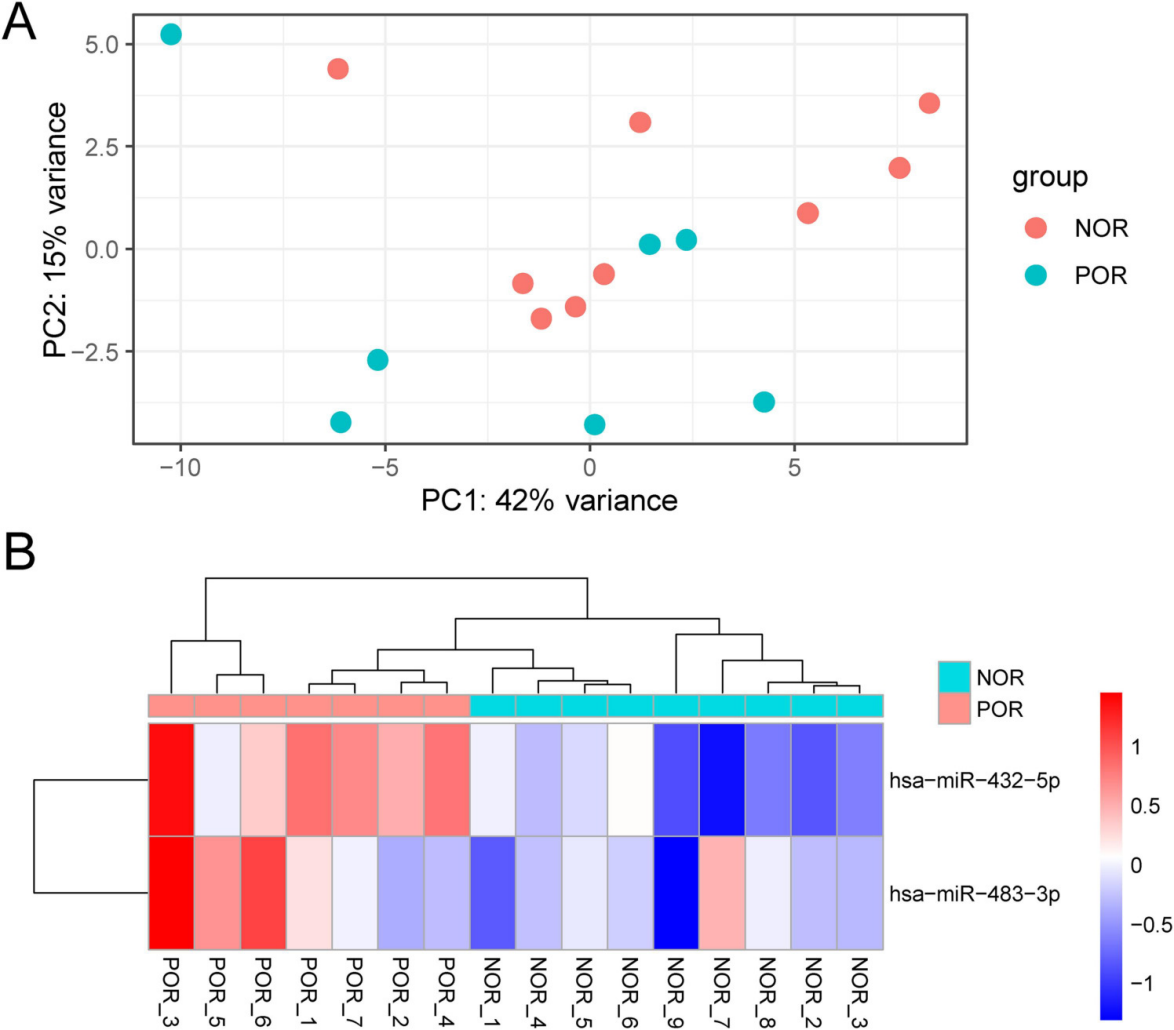
miRNAs regulated in G + CCs from women with weak ovarian responses. **(A)** PCA of the miRNA profile in G + CCs from NOR (red) and POR (blue) patients. **(B)** Heatmap showing differential expression of miR-432-5p and -483-3p in GCs from individual POR and NOR patients.

To identify miRNAs statistically differentially expressed in the G + CCs from PORs vs. NORs, we performed small-RNA-seq analysis on the 16 patients described above. PC analysis of these samples showed that the weak and normal responder samples segregated somewhat separately (Figure 5A), indicative of modest differences in miRNA expression between these two groups. Supplementary Table S4 shows the expression pattern of the 194 miRNAs expressed above the background threshold in the G + CC samples. Only 2 of these 194 miRNAs were significantly differentially expressed between the POR and NOR groups: miR-432-5p and miR-483-3p (*p* < 0.01). Figure 5B shows the expression of these 2 miRNAs in the individual patient samples. Both miRNAs exhibit reduced expression in G + CCs from most weak-responder patients. Both mRNAs have known roles that implicate them as candidates to influence ovarian responsiveness (see Discussion).

## Discussion

The major advance in the treatment of infertility over the past 40 years has been the introduction of IVF. However, this approach is not infallible. One of the major limitations of IVF is a low success rate for patients with weak responses to COH. In these poor-responding patients—which represent up to a quarter of all IVF patients—clinical pregnancy rates are low, and the psychological and interpersonal ramifications can be devastating. In this report, we provide evidence that specific classes of genes encoding physiologically relevant functions are dysregulated in GCs from patients with weak ovarian responses to COH.

A striking finding of our study was that 19 genes encoding proteins involved in the DNA-damage responses are statistically downregulated in POR G + CCs (Table 1). Their biochemical functions include maintaining genomic stability (BLM, DTL, FBXO5, MCM7, MCM10, PCLAF, and RAD51AP1), repairing DNA strand breaks (BRCA1, BRIP1, BTG2, POLQ, TOP2A, UBE2T, UHRF1, and WDR76), regulating DNA repair (BCL2), checkpoint regulation (BLM, CIP2A, and CDK1), and marking double-stranded DNA breaks (H2AX). Their dysregulation in weak-responder G + CCs is potentially important, as increased DNA damage due to reduced DNA repair is thought to contribute to ovarian ageing (63, 64). This is a significant issue, as follicles in the ovary can remain dormant for up to 50 years. While our data are consistent with the notion that one or more DNA repair mechanisms are impaired in POR G + CCs, it remains for future studies to directly test this.

Some of the DNA repair genes downregulated in weak-responder G + CCs are known to function in granulosa cells. For example, *BRCA1* has been shown to suppress granulosa cell apoptosis (46) as well as aromatase expression in granulosa cells (44); *BRCA1* also acts in granulosa cells to suppress estradiol levels systemically (65). *BLM* and *BTG2* are known to promote granulosa cell proliferation and survival (43, 48). *BCL2* has been linked to granulosa cell survival in follicles (41, 42).

All of the DNA damage-response genes that are statistically downregulated in POR G + CCs are linked with cancer. Most have been defined as oncogenes (*BCL2, CIP2A, CDK1, DTL, FBXO5, MCM7, MCM10, PCLAF, POLQ, TOP2A, RAD51, UBE2T*, and *UHRF1*), while the rest are tumor suppressor genes (*BLM, BRCA1, BRIP1, BTG2, H2AX*, and *WDR76*). As evidence for their importance in the ovary, many of these genes are associated with poor prognosis for ovarian cancer (Table 1). *PCLAF* and *MCM7* overexpression/amplification are specifically linked with most malignant form of ovarian cancer—high-grade serous ovarian carcinoma.

Another major observation of our study is that the genes downregulated in G + CCs from PORs are statistically enriched in numerous functional categories associated with cellular proliferation (Figure 4). In total, 52 of the 291 statistically downregulated genes in G + CCs from weak responders are known to be involved in proliferation. These downregulated genes encode classic cell cycle regulators (e.g., the cyclins CCNA2, CCNB1, and CCNB2, as well as CDCA8 and CDC20), kinases that regulate mitosis (e.g., AURKB, NEK2 and BUB1), and regulatory proteins that promote cell cycle progression (e.g., CDK1, CENPE, SGO1, and PLK1).

Some of the pro-proliferation genes that we found are statistically downregulated in POR G + CCs have been previously studied in GCs. For example, *BIRC5* encodes a multi-functional protein that promotes granulosa cell proliferation (66). *MKI67* encodes a positive regulator of the cell cycle widely used as a proliferation marker that has been found to be activated by the PI3K signaling pathway in porcine granulosa cells (67). *FOXM1* encodes a transcription factor that has been shown to promote granulosa cell proliferation in PCOS patients through activation of the WNT signaling pathway (68). *MYBL2* encodes another transcription factor that promotes granulosa cell proliferation and activates cell-cycle genes expression (69).

One explanation for POR G + CCs exhibiting low expression of batteries of pro-proliferation genes is these cells are poorly proliferative in PORs. In support, Han et al. reported that cultured POR GCs exhibit a trend towards a lower increase in cell number *in vitro* than do NOR granulosa cells (70). Further support comes from Seifer et al.*,* who found that the granulosa cells of women with a higher ovarian reserve had a higher proliferative index when compared to granulosa cells from women with a decreased ovarian reserve (71). If indeed POR GCs tend to be less proliferative than NOR G + CCs, the dysregulation of the large battery of proliferation genes that we observed in POR G + CCs is likely to be primarily a *consequence* of this compromised granulosa cell proliferation rate. However, this does not rule out that abnormal expression of one or more rate-limiting proliferation genes in POR patients also has a *causal* role in weak ovarian responses. For example, mutations or abnormal epigenetic modifications in key proliferation genes could be responsible for weak ovarian responses in some individuals.

Alterations in hormone signaling could also play a causal role in weak ovarian responsiveness. In this regard, we found that PORs exhibited a statistically significant reduction in *FSHR* expression (Supplementary Table S2). This is potentially important, as a functional FSHR and FSH signaling are critical for many aspects of ovarian responsiveness, including GC proliferation, GC differentiation, estradiol production, and maturation of ovarian follicles (32, 33). In agreement with our finding that PORs tend to express lower levels of *FSHR* than NORs, Cai et al. found that human granulosa cells from PORs had significantly lower levels of *FSHR* mRNA and FSHR protein compared to moderate and high responders (72). The downregulation of *FSHR* in PORs could explain the poor responsiveness of these patients to gonadotropins (72), which are not only critical for granulosa cells but other ovarian functions, including theca cell proliferation and steroid production (73). Another POR downregulated gene involved in steroidogenesis is *IHH*, which encodes a secreted signaling protein involved in theca cell recruitment, and is critical for steroid biogenesis in the female reproductive tract (61).

A key factor that determines ovarian reserve is programmed cell death (41). While the function “apoptosis” was not statistically enriched among regulated genes in POR vs. NOR granulosa cells, many genes encoding apoptosis-regulatory proteins were found to be statistically regulated. Strikingly, POR GCs downregulated a large group of apoptosis-inhibitory genes: *BCL2*, *BRCA1*, *BTG2*, *CIP2A, CDK1, DTL, FBXO5, MCM7, POLQ, RAD51AP1, UBE2T*, and *UHRF1* (42, 45, 46, 48–51, 53, 57, 59, 60) (Table 1). Interestingly, many of these genes also promote proliferation, and so the net potential effect of their negative regulation in poorly-responsive patients could be both reduced GC survival and proliferation. Potentially contributing to this reduced survival, we found that the pro-apoptotic genes, *ZNF185* (74) and *SLFN11* (75), are upregulated in POR G + CCs.

The finding that G + CCs from PORs have elevated expression of pro-apoptotic genes, coupled with their deficient expression of anti-apoptotic genes, raises the possibility that such G + CCs undergo higher rates of apoptosis than NOR GCs. In support, Seifer et al. found that women with high FSH levels (≥10 mIU/mL)—a predictor of POR—had fewer viable G + CCs per follicle and an increased percentage of cells undergoing apoptosis than women with low FSH levels (<6 mIU/mL), despite both groups displaying similar proliferation rates (76). Also potentially relevant to GC apoptosis, we found that the gene encoding the transcription factor, FOXM1, is downregulated in GCs from PORs. A prior study showed that downregulation of FOXO pathway signaling in G + CCs results in granulosa cell apoptosis and perturbed oocyte maturation (77). Given that FOXM1 is a key transcription factor regulated by the FOXO signaling pathway (78), this raises the possibility that FOXM1 downregulation in G + CCs could influence granulosa cell survival. We note, however, that whether increased granulosa cell apoptosis has a causal role in weak ovarian responses remains to be determined.

An unexpected finding was that “oocyte meiosis” and “progesterone-mediated oocyte maturation” are statistically enriched among downregulated genes in the POR group vs. the NOR group. The statistically downregulated “oocyte meiosis” genes are: *FBXO5, PTTG1, AURKA, CDC20, CCNB1, CCNB2, CDK1, MAD2L1, PLK1, SGO1,* and *PKMYT1*. The statistically downregulated “progesterone-mediated oocyte maturation” genes are: *AURKA, CDC25A, CCNA2, CCNB1, CCNB2, CDK1, KIF22, MAD2L1, PLK1*, and *PKMYT1*. Since these genes are only known to be involved in functions in oocytes, not G + CCs, the physiological significance of their dysregulation in G + CCs is not immediately obvious. Of note, these genes are not only involved in oocyte maturation but also associated with cell proliferation and thus they may be downregulated simply because G + CCs from weak responders are less proliferative. Others may encode proteins expressed in G + CCs that indirectly influence oocyte maturation. For example, the weak-responder downregulated gene, *PKMYT1*, encodes a membrane-associated serine-threonine kinase critical for oocyte progression. While PKYMT1 is only known to act directly in oocytes (79), it may also act indirectly on oocytes via expression on the surface of G + CCs.

Several of the genes statistically upregulated in GCs from weak ovarian-response patients have known roles in G + CCs, including *STC1*, *ANGPTL4,* and *IL1RL1* (Figure 3B). *STC1* encodes a glycoprotein hormone that has been reported to either inhibit or promote granulosa cell proliferation/survival depending on the species examined (36, 80). SCT1 also has other reported functions, including paracrine effects on granulosa cell differentiation that ultimately affect steroidogenesis (81). Given its complex effects, it is difficult to predict the consequences of the upregulation of SCT1 in weakly-responsive G + CCs. Another upregulated gene, *ANGPTL4*, also encodes a multi-functional secreted protein that, among other functions, inhibits granulosa cell proliferation (35). ANGPTL4 is a strong candidate to contribute to polycystic ovary syndrome (PCOS) symptoms, as it has effects on metabolism and its expression is elevated in G + CCs from PCOS patients (82). Intriguingly, *ANGPTL4* and *SCT1* expression are both known to be regulated by PPAR signaling pathways, as is another gene upregulated in GCs from weak-responsive patients: *PCK1* (83) (Supplementary Table S2). This leads to the hypothesis that one or more of the PPAR signaling pathways have a negative impact on oocyte production and folliculogenesis. In support, it is known that PPARs have numerous effects on ovarian functions (84).

Our finding that the cytokine receptor gene, *IL1RL1*, is statistically upregulated in weak ovarian-response patient G + CCs is interesting in light of this gene being dysregulated in cumulus cells from patients with diminished ovarian reserve (37). In addition, the gene encoding the cytokine that binds to IL1RL1, IL-33, is also upregulated in G + CCs from patients with diminished ovarian reserve (37). While there is limited knowledge concerning the effect of IL-33 on G + CCs, there is some evidence that this cytokine increases granulosa cell autophagy (85). In addition, IL-33 is known to promote the proliferation and survival of ovarian cancer cells (86).

We note that other studies have also identified genes in follicular fluid cells that are associated with ovarian dysfunction, including ovarian hypo-responsiveness, ovarian insufficiency syndrome, aging, and hormone receptor insufficiency. With regard to ovarian hypo-responsiveness, Choi et al. identified mis-regulated genes in preovulatory follicular fluid cells from hypo-responder patients undergoing IVF treatment (87). Using RNA-seq analysis, they identified several classes of mis-regulated genes, including ECM genes. Using single-cell (SC) RNA-seq analysis, they found that hypo-responders tend to have fewer ARGLU1^+^ and SEMA3A^+^ GCs, as well as fewer theca/stroma cells, raising the possibility that inter-patient variability in follicular cell composition is a contributor to differential gonadotropin sensitivity. Another study—Han et al.—used scRNAseq analysis to study follicular fluid from primary ovarian insufficiency patients (88). Their data suggested that granulosa cells from primary ovarian insufficiency patients undergo accelerated granulosa cell senescence caused by loss of the VEGFA–FLT1 signaling axis between monocytes and granulosa cells. Using a mouse model, Lazzaroni-Tealdi et al. studied ovarian aging and found that aging and superovulation dysregulate overlapping sets of granulosa cell genes, particularly those involved in hormonal responses and cell cycle control (89). These authors also found that granulosa cell transcriptional markers can prospectively predict an associated oocyte's early developmental potential (in mice), directly linking granulosa cell gene expression state to IVF outcomes. The studies above focused on protein-coding genes; other studies have examined the mis-regulation of non-coding RNAs in response to ovarian dysfunction (90). Specific long non-coding RNAs, circular RNAs, and miRNAs have been shown to be misregulated in GCs from patients with PCOS, premature ovarian insufficiency, and diminished ovarian reserve. These studies have obtained evidence that the PI3K/AKT, TGF-β, and Wnt signaling pathways contribute to ovarian dysfunctions, highlighting these pathways as being potentially useful for diagnosis and targets of therapy. Functional studies have begun to reveal the causal role of some of these pathways (91). Collectively, these studies have substantially advanced our understanding of the molecular basis of ovarian dysfunction. Our study builds on this foundation by providing an unbiased, whole-transcriptome analysis, encompassing both mRNAs and miRNAs, of G + CCs gene expression differences between PORs and NORs undergoing COH for IVF. Our study also identified candidate biomarkers and dysregulated pathways that may underlie weak ovarian responses and could potentially serve as targets for improving IVF outcomes.

We also identified two miRNAs that are statistically downregulated in G + CCs from poorly-responsive patients: miR-432 and −483. The dysregulation of miR-483 is intriguing, as it is known to influence several events in the ovary, including cell proliferation, apoptosis, and differentiation in the ovary (92, 93). miR-483 is also dysregulated in PCOS (94, 95). Given that miR-483 promotes proliferation (96), its reduced level in POR patients may exert a causal role in their weak ovarian responses by reducing granulosa cell proliferation. The other miRNA statistically downregulated in POR G + CCs—miR-432—is encoded in the DKL1-DIO3 imprinted region (14q32). Like miR-483, miR-432 regulates proliferative responses; its functions also extend to metabolic and inflammatory pathways (97). Thus, the dysregulated expression of miR-432 in POR G + CCs has the potential to influence ovarian responses by several different mechanisms. We suspect that we only detected two miRNAs statistically dysregulated in POR G + CCs because of the limited power of our small RNA-seq dataset. Future studies will be required to delineate the full extent of miRNA dysregulation associated with PORs. Nonetheless, we regard our discovery of two mi-regulated miRNAs with known activities relevant to G + CCs dysfunction as intriguing and potentially clinically relevant.

Caveats with our study include the inherent biological variability of human clinical samples and the limited sample size available for our transcriptomic analysis. Accordingly, the RNA-seq findings should be interpreted as exploratory and hypothesis-generating rather than definitive at a population level. Larger prospectively powered studies will be necessary to validate the molecular signatures identified. In addition, future studies incorporating prospectively collected samples with complete clinical annotation and outcome follow-up will be essential to establish stronger links between granulosa cell transcriptomic profiles and clinically meaningful IVF outcomes.

In summary, our study (i) provides new avenues for researching the pathophysiologic ovarian processes underlying subfertility and infertility, (ii) identifies potential biomarkers to diagnose sub-optimal ovarian responses, and (iii) offers future targets for infertility treatments.

## Data Availability

The datasets presented in this study can be found in online repositories. The names of the repository/repositories and accession number(s) can be found in the article/Supplementary Material.

## Supplementary material

Supplementary Figure 1 Clinical and endocrine characteristics of women retrieving 6–10 oocytes exhibited no significant differences from poor responders (≤5 oocytes). Parameters shown include: (A) Antral follicle count (AFC), (B) Estradiol level (pg/mL) on day of hCG trigger, (C) follicle-stimulating hormone (FSH), (D) Anti-Mullerian hormone (AMH), (E) Age, and (F) Body mass index (BMI) (kg/m^2^).

Supplementary Figure 2 RNA-sequencing workflow performed on 16 samples.

Supplementary Table 1 Patient Baseline Characteristics.

Supplementary Table 2 List of differentially expressed RNAs comparing PORs vs. NORs.

Supplementary Table 3 List of “core genes” defined by the weighted gene co-expression network analysis that are differentially expressed between PORs vs. NORs.

Supplementary Table 4 List of differentially expressed miRNAs comparing PORs vs. NORs.
