# Supplementary figures and images for: Identification of genes differentially expressed in granulosa cells from women with high vs. low ovarian responsiveness
Source: Front Reprod Health. 2026 Jun 10;8:1819763. doi: 10.3389/frph.2026.1819763 (PMC13290879; doi:10.3389/frph.2026.1819763)

Supplementary Figure 1

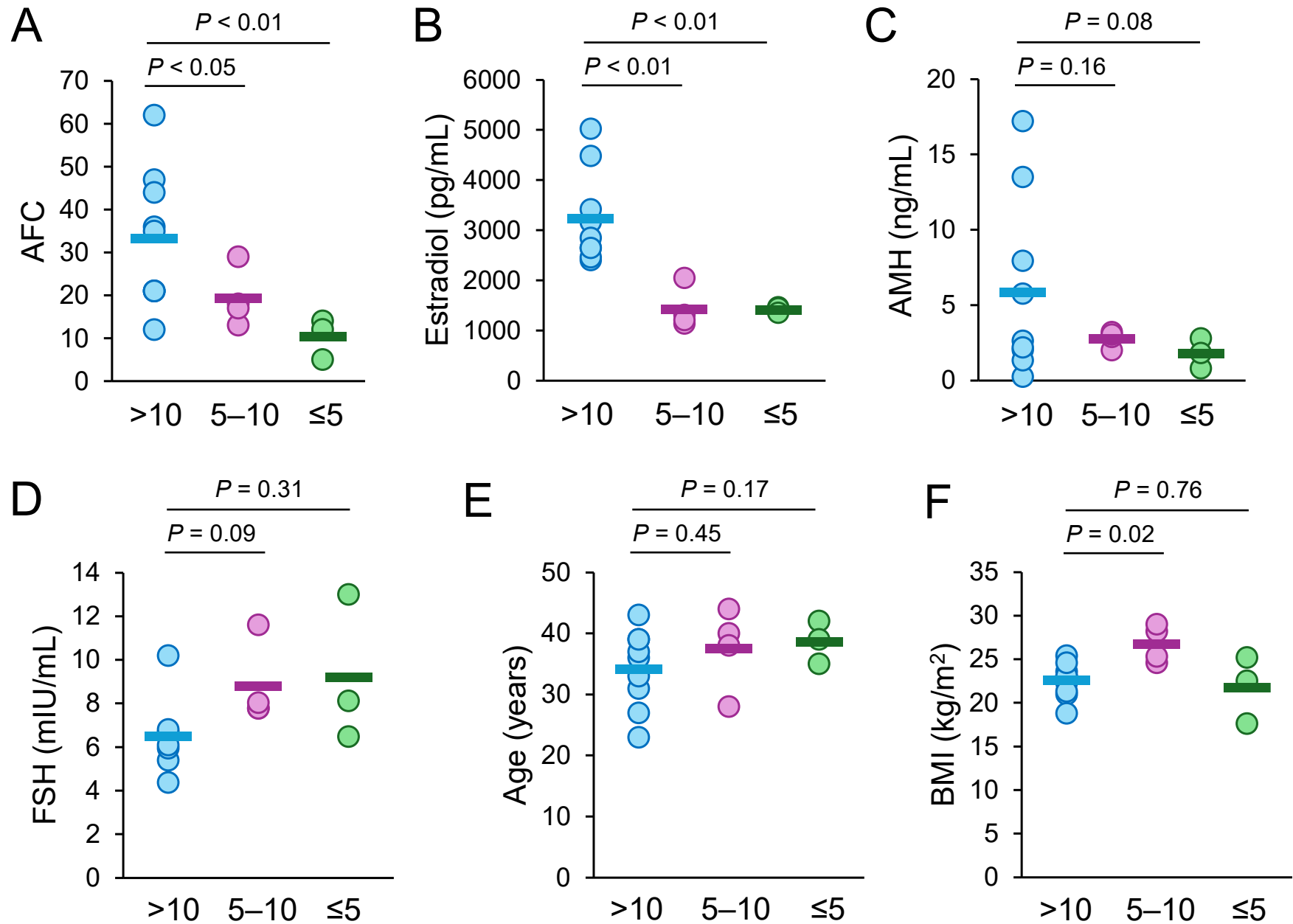

# Supplementary Figure 2

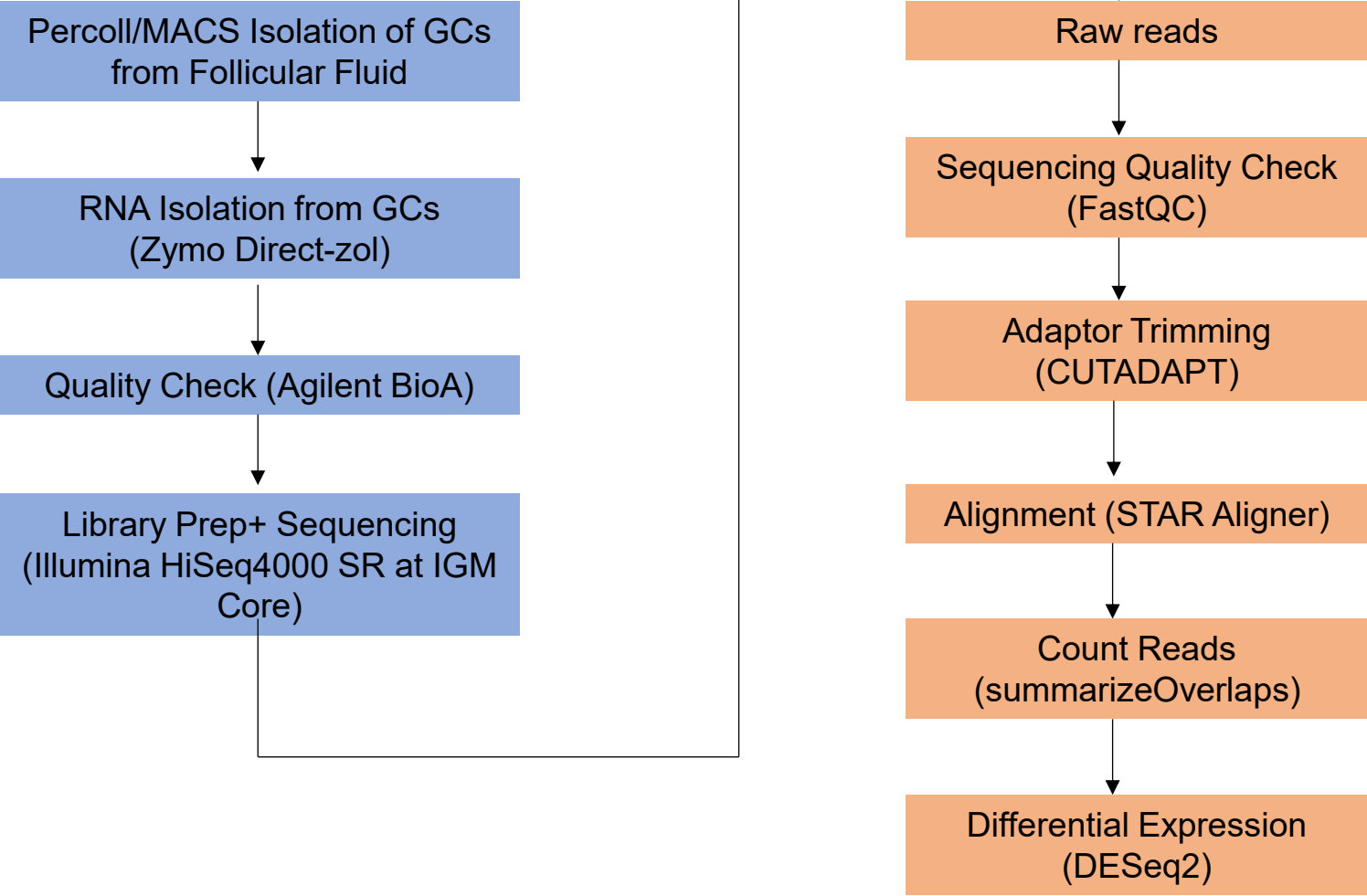

Supplement: Supplementary file 2 [file Datasheet1.pdf]
